# Thrombectomy and Extraction of Patent Foramen Ovale Occluder With Repair of Atrial Septum: A Minimally Invasive Approach

**DOI:** 10.1016/j.cjcpc.2026.01.001

**Published:** 2026-01-08

**Authors:** Mathieu Rheault-Henry, Meera Patel, Lin Rui Guo

**Affiliations:** a Division of Cardiac Surgery, Department of Surgery, Western University, London, Ontario, Canada; b Schulich School of Medicine and Dentistry, Western University, London, Ontario, Canada

**Keywords:** patent foramen ovale, stroke, thrombectomy, mini thoracotomy, atrial septal defect repair

**Percutaneous closure of a patent foramen ovale is an established strategy for secondary stroke prevention but still carries some risks, including device embolization, thrombosis, and recurrent embolic events. We report the case of a 58-year-old man who developed multiple ischemic strokes 4 years after implantation of a Gore patent foramen ovale occluder due to late device-related thrombosis. He underwent a surgical thrombectomy, partial occluder extraction, and atrial septal repair through a right anterior minithoracotomy with peripheral cannulation. This approach resulted in a rapid recovery and short hospital stay, underscoring the potential role of minimally invasive techniques in managing select device-related complications.**

## Case

A 58-year-old man with hypertension and remote smoking was referred for thrombectomy and extraction of a Gore patent foramen ovale (PFO) occluder after experiencing recurrent thromboembolic strokes attributed to late device thrombosis. His initial presentation occurred in early 2019 with sudden right-sided hemiplegia and dysarthria, which improved after treatment with tissue plasminogen activator (tPA). After 2 weeks of rehabilitation, he was discharged without residual deficits and placed on lifelong aspirin therapy.

**Figure 1:**
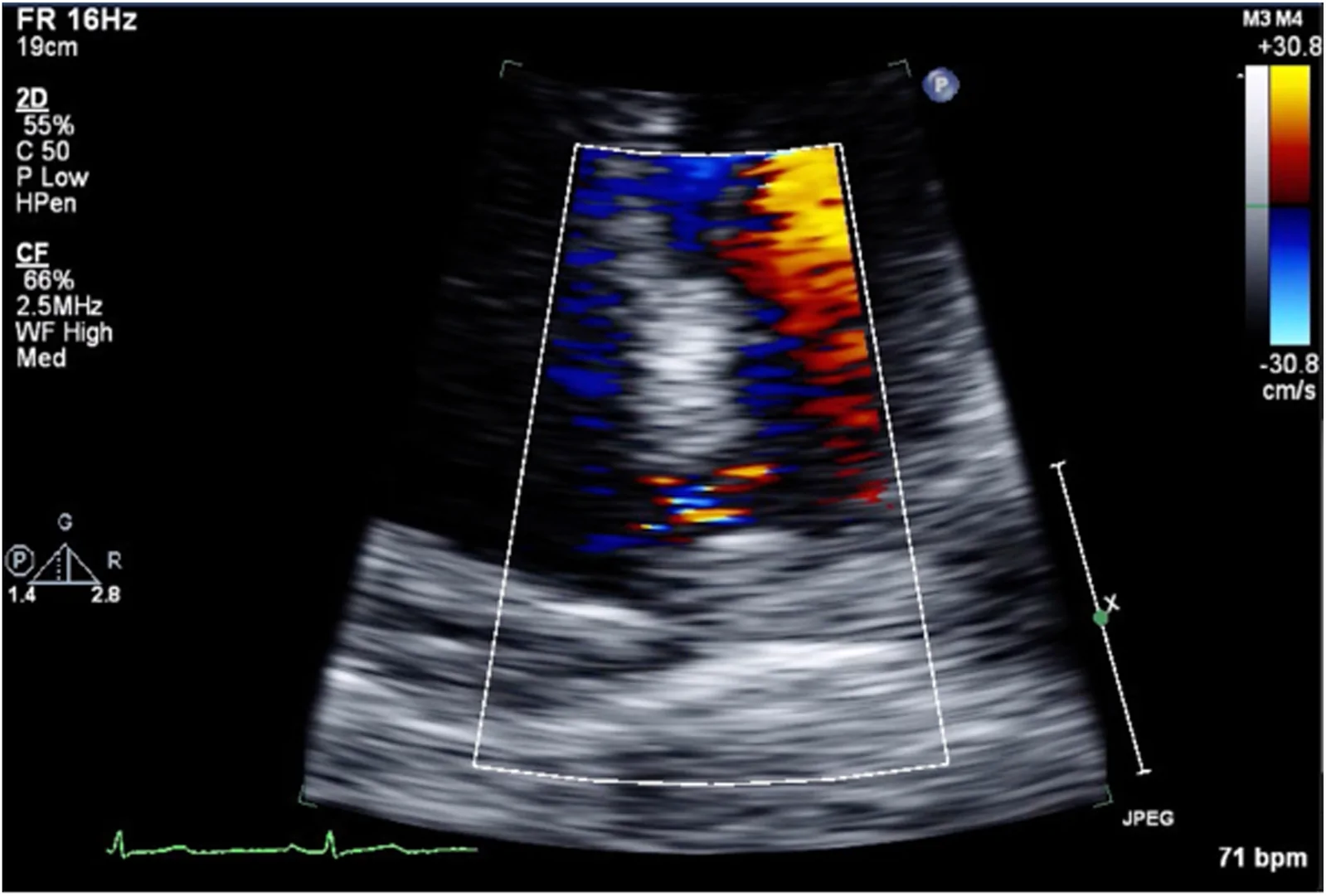
Transthoracic echocardiogram demonstrating patent foramen ovale with transient right-to-left shunt on zoomed-in apical 4-chamber view.

Five months later, he developed recurrent left-sided hemiplegia and dysarthria. Cerebral computed tomography demonstrated multiple right frontotemporal infarcts, consistent with recurrent embolic phenomena. Repeat tPA was administered with clinical improvement. Hematologic evaluation revealed no hypercoagulable disorder. Transthoracic echocardiogram (TTE) with a bubble study demonstrated a small-to-moderate PFO with transient right-to-left shunting (Fig. 1). There was no evidence of pulmonary embolism on computed tomography, nor any significant tricuspid regurgitation or Chiari network on TEE. He exhibited no features of pulmonary hypertension or Eisenmenger syndrome. With this in mind, PFO closure was recommended, and clopidogrel was added to his antiplatelet regimen.

A 30-mm Gore septal occluder was successfully deployed, with no residual shunt seen after the procedure. After 3 months of dual antiplatelet therapy, he was transitioned to lifelong aspirin. A repeat bubble study 6 months later confirmed complete closure.

**Figure 2:**
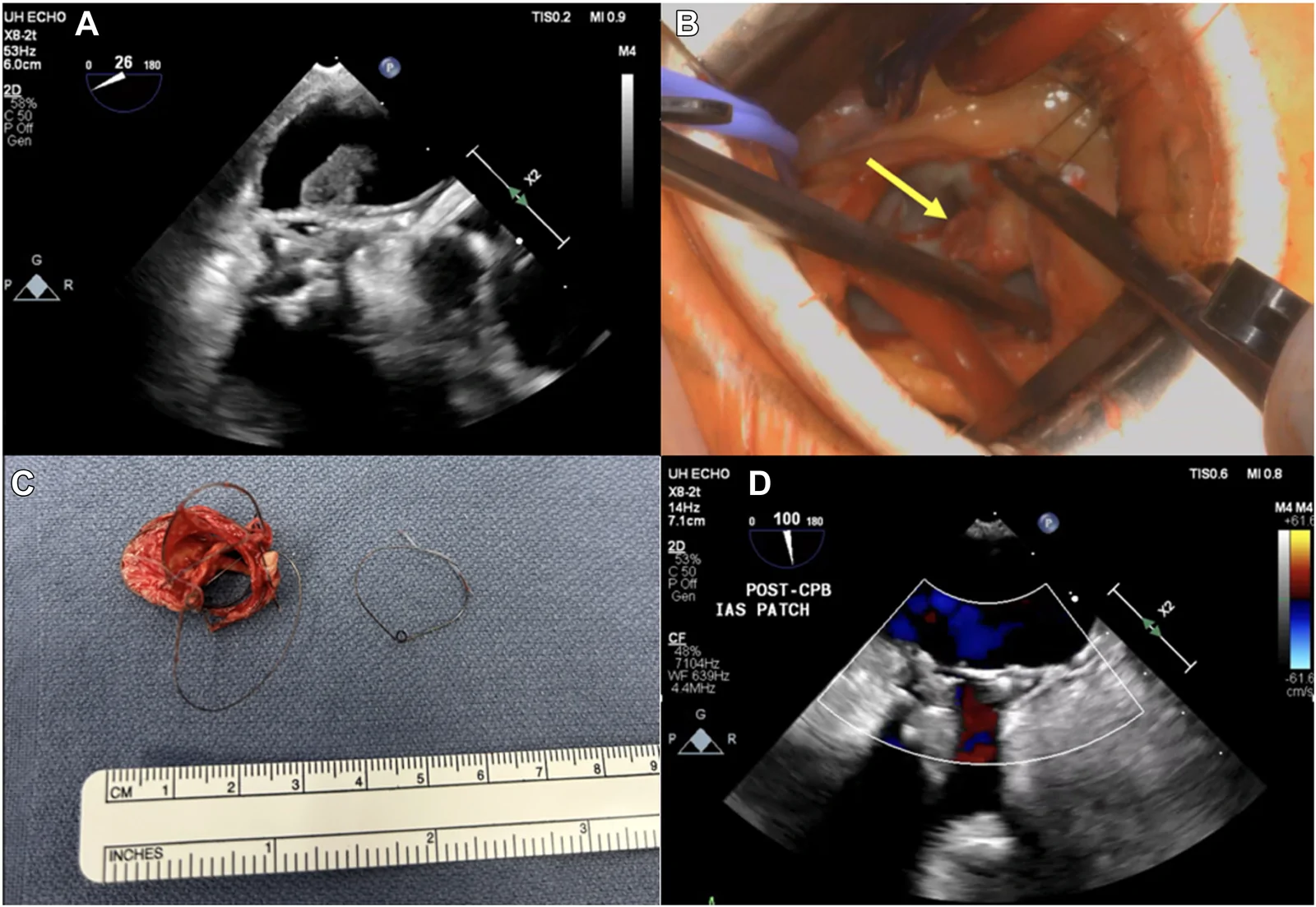
(**A**) Left atrial thrombus attached to the Gore septal occluder. (**B**) Intraoperative view of the left atrium with a 14 × 12 mm thrombus visualized on the septal occluder. (**C**) Patent foramen ovale (PFO) occluder after explantation. (**D**) Repaired interatrial septum with a bovine pericardial patch with no residual PFO.

In mid-2023, he experienced a third ischemic event—a left middle cerebral artery stroke. He was again treated with tPA, with mild residual right-sided hemiparesis. No atrial fibrillation was detected. The following day, TEE revealed a 9 × 9 mm mobile thrombus attached to the left atrial surface of the occluder, adjacent to the exposed central metal pin connecting the device’s discs (Fig. 2A). The occluder remained well seated without residual shunt or left atrial appendage thrombus. He was referred for surgical thrombus removal and device explantation. Cerebral imaging did not demonstrate hemorrhagic transformation after thrombolysis, allowing for safe progression to surgery the next morning.

Urgent minimally invasive extraction was performed through a 5-cm right anterolateral minithoracotomy. This well-described approach for atrial septal defect closure provided excellent access while avoiding the morbidity associated with median sternotomy. Femoral arterial and venous cannulation enabled initiation of cardiopulmonary bypass. A right lateral pericardiotomy was performed to access the heart. An aortic cardioplegia cannula was inserted, and del Nido cardioplegia was administered for myocardial arrest. Left atriotomy revealed a highly mobile thrombus adherent to the device’s protruding metal pin, which had dislodged from the inner disc. The thrombus, metal pin, and associated left atrial disc components were sharply dissected and removed (Fig. 2B and C). The resulting atrial septal defect was reconstructed with a 2 × 2 cm bovine pericardial patch using running 4-0 Prolene sutures.

A subsequent right atriotomy permitted visualization of the right atrial disc, which was fully endothelialized and calcified. Because further dissection risked disrupting the atrial septum, and because the right atrial disc was fully endothelialized, immobile, calcified, and posed no additional embolic risk, it was left *in situ*.

Postoperative TEE confirmed an intact interatrial septum with no shunt (Fig. 2D). The patient recovered uneventfully and was discharged on postoperative day 4. By 6-week follow-up, he had resumed full activity and remains clinically well without recurrent neurologic events, shunts, or device-related complications at 2-year follow-up.

## Discussion

PFO is a well-recognized risk for paradoxical embolism and ischemic stroke, with lifetime stroke risks estimated between 6.2% and 21.4%.^1^ In our patient, the mechanism of paradoxical embolism was likely explained by transient right-to-left shunting, caused by transient elevations in right atrial pressure rather than fixed hemodynamic abnormalities. Under normal conditions, left atrial pressure exceeds right atrial pressure by a few millimeters of mercury. However, maneuvers such as Valsalva, coughing, supine positioning, or normal respiratory variation can temporarily reverse this gradient. In the presence of a PFO, these brief increases in right atrial pressure can facilitate paradoxical embolization.

In appropriately selected patients with cryptogenic stroke, PFO closure provides superior protection against recurrent events compared with medical therapy, particularly in patients younger than 60 years.^2^ Percutaneous PFO closure is considered a safe and effective approach, but related complications such as device thrombosis, device embolization, and residual shunting may occur.^3^ Foreign material in the left atrium may act as a nidus for thrombus formation. Procedural complications occur in approximately 0.8% of cases, and device-related thrombosis in roughly 2%, most frequently within the first month.^4^ Contemporary antithrombotic regimens have reduced this risk to approximately 0.3% at 1 year.^5^ However, late thrombus formation, as demonstrated in this case, although rare, remains clinically relevant, and providers should be aware of this.

In this case, thrombus formation originated from the exposed metal pin of the occluder’s left atrial disc. The pin is designed to anchor the disc within the interatrial septum and is not intended to protrude into the left atrial cavity. However, when exposed to the systemic circulation, it may serve as a prothrombotic surface and act as a nidus for thrombus formation. In patients with device-related thromboembolism, surgical management is indicated and includes thrombus removal, device extraction when feasible, and reconstruction of the interatrial septum.

Median sternotomy remains the conventional approach for device explantation and is the historic standard for atrial septal defect closure. However, right minithoracotomy approaches have been well established in the literature, demonstrating reduced blood loss, fewer infections, and a shorter recovery. Leveraging these advantages, our minimally invasive approach enabled complete thrombus removal, targeted extraction of nonendothelialized device components, and secure atrial septal reconstruction while minimizing surgical trauma. To our knowledge, this is among the first reported applications of minimally invasive atrial septal repair and partial occluder explantation for late PFO device thrombosis.

This case underscores the importance of recognizing that device-related complications can occur well beyond the early postimplantation period, necessitating ongoing awareness and patient counseling. It further highlights the educational relevance of understanding occluder design, identifying atypical device-related complications, and applying minimally invasive surgical principles.

Although our experience demonstrates the feasibility of minimally invasive extraction, further studies are warranted to evaluate long-term outcomes, refine patient selection, and establish standardized management strategies for late device-related thrombosis.

**Novel Teaching Points**

• Late thrombosis of patent foramen ovale occluder devices can occur years after implantation and cause recurrent embolic stroke despite antiplatelet therapy, warranting further echocardiographic evaluation.
• Exposed occluder components may serve as a nidus for thrombus formation and late embolic events.
• Minimally invasive right minithoracotomy enables safe thrombectomy, selective device extraction, and atrial septal repair, providing an alternative to sternotomy in selected cases.
